# Nutrition and physical activity practices in family day care: A cross‐sectional survey of Australian family day care educators

**DOI:** 10.1002/hpja.913

**Published:** 2024-08-19

**Authors:** Georgie Tran, Erin Kerr, Bridget Kelly, Sarah T. Ryan, Jennifer Norman, Megan Hammersley, Cecilia Vuong, Karen Wardle, Anthony Okely

**Affiliations:** ^1^ School of Health and Society, Faculty of the Arts, Social Sciences and Humanities University of Wollongong Wollongong Australia; ^2^ Health Promotion Service, Sydney Local Health District Camperdown Australia; ^3^ Health Promotion Service Illawarra Shoalhaven Local Health District Warrawong Australia; ^4^ Health Promotion Service South Western Sydney Local Health District Liverpool Australia

**Keywords:** children, family day care, Munch & Move, nutrition, physical activity, screen time

## Abstract

**Issue Addressed:**

*Munch & Move* is a New South Wales (NSW) Ministry of Health program offering family day care (FDC) educators training to support children's healthy behaviours. This study examined educators' nutrition, physical activity and screen time practices and relationships between *Munch & Move* training and professional development (PD) on these practices.

**Methods:**

NSW FDC educators (*n* = 186) completed an online survey from July 2020–June 2021. Differences between groups based on *Munch & Move* training (trained; not trained) and PD (those who completed PD ≥1 time per year; those who completed PD <1 time per year or never) were tested using bivariate analyses.

**Results:**

A significantly higher proportion of educators trained in *Munch & Move* offered information to families regarding food serving sizes, nutrition policies, and children's physical activity and screen time. Over one‐third in both groups did not meet the guideline of no screen time for children under 2 years old. Compared with those who completed PD ≥1 time per year, a significantly higher proportion of educators who completed PD <1 time per year or never did not provide families with nutrition guidelines or resources.

**Conclusions:**

Educators trained in *Munch & Move*, and those who completed PD more frequently, demonstrated better nutrition, physical activity and screen time practices in several areas.

**So What?:**

This study demonstrated benefits of the *Munch & Move* program, implemented with support from Local Health District health promotion officers, and highlighted key areas for improvement in healthy practices in FDC.

## 1 BACKGROUND

The New South Wales (NSW) Ministry of Health's First 2000 Days Framework highlights the importance of a child's first 2000 days for physical, cognitive, social and emotional development and health.^1^ Promoting good nutrition and healthy levels of movement behaviours (physical activity, sedentary behaviour and sleep) are important in this framework. In Australia, approximately 25% of children aged 5–14 years and 20% of children aged 2–4 years are living with overweight or obesity.^2^, ^3^ Children living with overweight or obesity have an increased risk of chronic disease and premature mortality later in adulthood.^3^ Prevention of childhood obesity is reflected in government policies, including the NSW Healthy Eating and Active Living Strategy^4^ and there is some evidence of cost‐effectiveness of service based prevention strategies.^5^ In Australia, almost one third of the total burden of childhood obesity is due to modifiable risk factors such as poor nutrition and physical inactivity.^6^

Family day care (FDC) services are an important setting to promote healthy eating behaviours and physical activity in young children.^7^ In Australia, FDC is a home‐based model of care and is part of the early childhood education and care (ECEC) sector. Out of *n* = 1 419 380 Australian children who regularly attend child care services, *n* = 72 690 (5.1%) attend FDC.^8^ Overseas equivalents of FDC include ‘home‐based care’ in New Zealand, ‘pedagogical care’ in Sweden, and ‘family child care’ in Canada and United States of America.^9^ Children attending FDC can have their food provided from home, their food provided by FDC educators or have food provided by both educators and from home.^10^ However, previous research indicates that food and physical activity environments in FDC can be improved to better support healthy behaviours.^7^ In a study conducted in South Australia, FDC educators were asked about their nutrition‐related practices including food provided by parents or themselves. While there was agreement amongst respondents that nutrition promotion was relevant to their jobs, around half of respondents did not feel confident in addressing food quality issues with parents, and only one‐quarter of respondents felt confident in addressing inappropriate food and drink choices with parents.^11^ Recent findings from an audit of healthy eating and physical activity practices in NSW FDC services found that children attending FDC were provided with excess discretionary foods and were not meeting recommendations for vegetables, dairy, meat/meat alternatives, or wholegrains. Furthermore, less than half of the children were participating in recommended levels of physical activity.^12^

*Munch & Move* is a NSW Ministry of Health administered program offering educators who work in early childhood education and care services training and resources to support the development of healthy eating and physical activity behaviours in young children. Implementation support is provided from Local Health District health promotion officers.^13^ The *Munch & Move* program focuses on six key messages related to healthy eating and physical activity—encourage and support breastfeeding, choose water as a drink, choose healthier snacks, eat more fruit and vegetables, get active each day and turn off the screen and get active. As part of the program, practical resources are provided for early childhood educators, such as fact sheets to communicate with families and resource manuals, are available. In NSW, FDC service providers are responsible for the operations of early childhood education and care for educators who are registered with their service. This includes recruiting and training educators, family and educator registrations, and providing ongoing support towards the provision of quality education and care.^14^

The *Munch & Move* program provides FDC service providers with training and support through the program, who in turn support their FDC educators to adopt health‐promoting practices.^15^ Training is offered free to ECEC educators and service leaders. For FDC, the support is provided to the service provider coordination unit. Training and resources provided include professional development (PD) training, resources such as family fact sheets and manuals, support to develop and implement healthy policies and practices, practical tips and ideas and contact with Local Health District health promotion officers for additional advice and support.^13^ The *Munch & Move* eLearning training has been implemented since April 2019 and is a self‐paced program that includes videos, interactive activities and reflective questions which assist educators to embed healthy eating and physical activity practices in their FDC service. Approximately 88% of centre based ECEC services are currently participating in the program, indicating the program's reach and demonstrating the awareness of and experience with the program from a range of childcare settings.^15^ This suggests the scalability of the *Munch & Move* program and indicates it is worthwhile investigating the program's implementation in FDC settings.

ECEC educators have identified that lack of staff training and lack of confidence when talking about nutrition are barriers to their nutrition knowledge.^16^ As research indicates that educators' knowledge and attitudes are barriers to providing healthy eating environments,^7^ PD opportunities focusing on improving nutrition knowledge and confidence in talking about nutrition may be beneficial for educators to enhance their feeding practices and create a supportive mealtime environment. In addition, a study examining the physical activity environment of children attending FDC identified that educator PD on physical activity led to better physical activity levels in children.^17^

The National Quality Framework (NQF) specifies regulations and learning frameworks that ECEC services, including FDC, are required to meet or implement, and encompasses the National Quality Standard (NQS). The NQS acts as a benchmark for ECEC services and outlines seven quality areas that are important outcomes for children. Meeting of exceeding these quality areas is an expectation of the Education Board when assessing suitability of services providing care to children.^18^ The *Munch & Move* program encourages and supports services to implement healthy eating and physical activity strategies for children in their care, aligning specifically with Quality Area 2 of the NQS.^13^

To better facilitate future health promotion interventions in FDC, a comprehensive understanding of current nutrition and physical activity practices in FDC is needed, including how these practices may be associated with the *Munch & Move* program. This study examined the current nutrition, physical activity and screen time practices of educators in NSW FDC and explored relationships between *Munch & Move* training and PD on these practices. We hypothesised that educators trained in the *Munch & Move* program, and those who completed PD more frequently, would display better nutrition, physical activity and screen time practices than those who had not received the training or who had not completed PD.

## 2 METHODS

### 2.1 Study design and sample

This research was a cross‐sectional study using an online survey and used the STROBE checklist for reporting. The survey was piloted by members of the project Advisory Group, including Local Health District health promotion officers and two staff members from NSW Family Day Care Association (the industry peak organisation). The collection and management of study data were facilitated through the REDCap's electronic data capture tools, including the REDCap Mobile Application,^19^ hosted at the University of Wollongong in Australia.

The study was approved by the University of Wollongong and Illawarra Shoalhaven Local Health District Health and Medical Human Research Ethics Committee (HREC/18/WGONG/13). Data were collected between July 2020 and June 2021. A convenience sample of FDC educators across NSW was recruited for this study. Online consent was obtained from educators who were willing to participate. Invitations to participate were sent through multiple channels. First, all FDC service providers in NSW were emailed an invitation for their educators to participate. The email addresses for NSW service providers were obtained from The Australian Children's Education & Care Quality Authority database which is publicly available. Second, South Western Sydney Local Health District and Illawarra Shoalhaven Local Health District health promotion services were asked to forward invitations to participate to their FDC contacts. In addition, NSW Family Day Care Association emailed their members which included both service providers and educators.

### 2.2 Measures

The survey was based on the Nutrition and Physical Activity Self‐Assessment for Child Care surveys.^20^ The survey was adapted to be in line with Australian Dietary Guidelines^21^ and *Munch & Move* best practices.^13^

*Munch & Move* best practices for nutrition include:

- Breastfeeding alone is recommended until around 6 months of age. After that, solids can be introduced while continuing breastfeeding up to 12 months of age or longer. Educators can encourage and support mothers who wish to breastfeed and provide information to all families on appropriate introduction to first foods and drinks.
- Young children should be encouraged to drink plenty of water every day. Milk is also an ideal drink as it is an important source of protein, calcium and vitamin D.
- Children should be provided with a variety of nutritious foods from the five food groups each day. Fruits and vegetables can be served in a variety of ways to engage children.
- Snacks should be based on healthy, filling foods such as vegetables, wholegrain cereal‐based foods, fruits and dairy products.

*Munch & Move* best practices for physical activity include:

- Children should be active every day in as many was as possible. For babies (birth to 1 year of age), physical activity should be encouraged, particularly with supervised floor‐based play.
- For toddlers (1–2 years of age) and preschool aged children (3–5 years of age), they should be physically active every day for 90 min or more per day.
- Activities should be developmentally appropriate, fun, and varied, promoting motor skills and physical competence.

*Munch & Move* best practices for screen time include:

- Sedentary screen time is not recommended for children younger than 2 years of age.
- For children 2–5 years of age, sedentary screen time should be limited to less than 1 h per day.

Terminology specific to the ECEC sector were also added. The survey comprised 47 questions that were split across three sections. Questions included single‐answer, multiple‐answer and drop down selection questions. The survey was expected to take 30 min to complete. FDC educators reported their demographic details regarding their work experience, qualifications, details of the provision of care for children enrolled through FDC and ages of children cared for.

Questions regarding nutrition practices collected information on:

- Food provision, for example, are families provided with nutritional guidelines or resources regarding recommended foods and drinks to pack in their child's lunchbox?
- Eating environment, for example, how often are televisions or videos turned on during meal or snack times?
- Feeding practices, for example, how often do you praise children for trying new or less preferred foods?
- Education and development, for example, how often do you complete PD on child nutrition?

Questions regarding physical activity practices collected information on:

- Physical activity, for example, what is the amount of time provided for children's indoor and outdoor physical activity each day?
- Outdoor playtime, for example, how often do you provide time for outdoor play?
- Outdoor play environment, for example, what portable play equipment is available and in good condition for children to use outdoors?
- Education and development, for example, how often do you complete PD on children's physical activity?

Questions regarding screen time practices collected information on:

- Screen time, for example, what is the amount of sedentary screen time allowed in your FDC each week?
- Education and development, for example, how often do you complete PD on children's screen time?

### 2.3 Data analyses

Data were analysed using the Statistical Package of the Social Sciences Software version 22 (Armonk, NY: IBM Corp.). Descriptive statistics were used to summarise the data. Differences between groups based on *Munch & Move* training background of educators (trained or not trained) and PD background of educators (those who completed PD ≥1 time per year or those who completed PD <1 time per year or never completed PD) were assessed using bivariate analyses; Chi‐squared tests and Fisher's exact tests. Significant differences were identified at *p* < .05. Clustering by FDC service providers was considered, however this was not possible as many FDC service providers were represented by only one of their educators. Although data on socio‐economic status and geographical location were collected, statistical analyses could not be performed as all participants were from high socio‐economic areas according to the Australian Bureau of Statistics Socio‐Economic Indexes for Areas.^22^

## 3 RESULTS

**FIGURE 1:**
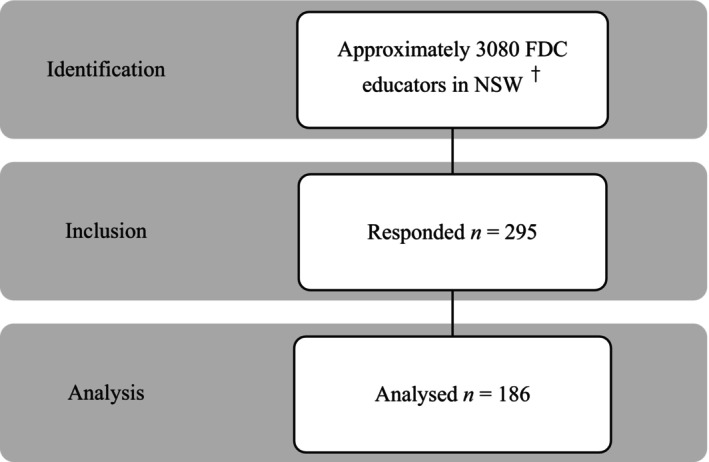
Flow chart of participants recruited. ^†^Approximate number of FDC educators in NSW according to the Family Day Care Association Sector Profile in June 2022. The statistics may not be accurate due to multiple services forced to shut down after a government audit in the past few years.^23^, ^24^

A total of 295 educators responded to the survey, of which 186 (63%) completed the survey and were included in the final data analysis. Figure 1 depicts a flow chart of the participants recruited. Of the excluded responses, 27 were duplicates and 82 were incomplete (most of these (93%) had at least two out of three sections incomplete). As such, no analyses were possible. The duplicates occurred during a second round of recruitment in the following year in which 27 educators re‐completed the survey.

The demographic characteristics of the final sample is shown in Table 1. All participants were from high socio‐economic areas according to the Australian Bureau of Statistics Socio‐Economic Indexes for Areas.^22^ English was the most common language spoken at home (69%). Almost one‐third of educators (31%) spoke a language other than English as their main home language.

**TABLE 1:** Demographic characteristics of participants (*n* = 186).

Demographic characteristics	*n*	%
Language spoken most at home		
English	128	69
Hindi	11	5.9
Tamil	7	3.8
Punjabi	6	3.2
Other^a^	34	18
Years worked in the Early Childhood Education and Care (ECEC) sector		
Less than 1 year	9	4.8
1–5 years	36	19
6–10 years	45	24
11–15 years	29	16
16–20 years	32	17
21–25 years	16	8.6
26+ years	19	10
Years worked as a family day care educator		
Less than 1 year	15	8.1
1–5 years	63	34
6–10 years	44	24
11–15 years	23	12
16–20 years	23	12
21–25 years	10	5.4
26+ years	8	4.3
Highest early childhood education qualification completed		
Certificate III in ECEC	86	46
Diploma in ECEC	80	43
Undergraduate University Degree	16	8.6
Postgraduate University Degree	2	1.1
Other^b^	2	1.1
In a usual week, the number of days that care is provided for children enrolled through family day care		
1 day	2	1.1
2 days	3	1.6
3 days	23	12
4 days	46	25
5 days	104	56
More than 5 days	8	4.3
Based on a usual week, the number of hours on an average day that care is provided for children enrolled through family day care		
4 or less hours a day	2	1.1
Between 5 and 6 h a day	3	1.6
Between 7 and 8 h a day	48	26
Between 9 and 10 h a day	107	58
11 or more hours a day	26	14
Ages of children that attend family day care (multiple responses allowed)		
Under 12 months	60	32
2 years	154	83
3 years	151	81
4 years	121	65
5 years	73	39
6 years	41	22
7 years and above	61	33

^a^ Participants who selected ‘other’ wrote the following in the free‐text box: Afrikaans *n =* 1, Vietnamese *n* = 1, Spanish *n =* 1, Marathi *n* = 1, Samoan *n* = 1, Bengoli *n* = 3, Arabic *n* = 3, Bangla *n* = 6, Telugu *n* = 5, Dari and Persian *n* = 1, French *n* = 1, Sinhalese *n* = 2, Korean *n* = 1, Nepali *n* = 1, Mandarin *n* = 3, Farsi *n* = 1, Hakha Chin *n* = 1, Aboriginal *n* = 1.

^b^ Participants who selected ‘other’ wrote the following in the free‐text box: Cert IV Children's Services *n* = 1, Associate Diploma of Social Sciences Child Studies *n* = 1.

### 3.1 Nutrition practices

The nutrition practices examined included food provision, eating environment, feeding practices and education and development. Table 2 compares nutrition practices between educators trained in *Munch & Move* eLearning relating to child nutrition (*n* = 98) and those who were not trained (*n* = 88). A significantly higher proportion of trained educators offered information to families regarding serving sizes for children compared to those who were not trained (69% vs. 55% respectively, *p* = .04). A higher proportion of those who were trained in *Munch & Move* offered information on their services' policies on child nutrition compared to those who were not trained (78% vs. 61%, *p* = .02). A significantly smaller proportion of trained educators responded that they rarely or never led planned healthy eating learning experiences compared to those who were not trained (1% vs. 9%, *p* = .01).

**TABLE 2:** Nutrition practices by educators trained in *Munch & Move* eLearning relating to child nutrition.

	All educators (n = 186) *n* (%)	Educators trained in Munch & Move eLearning (*n* = 98) *n* (%)	Educators not trained in Munch & Move eLearning (*n* = 88) *n* (%)	*p*‐Value^a^
Information offered to families on child nutrition (multiple responses allowed)				
Food and beverage recommendations for children	161 (87)	67 (68)	74 (84)	.01
Serving sizes for children	116 (62)	68 (69)	48 (55)	.04
Importance of variety in the child's diet	140 (75)	79 (81)	61 (69)	.08
Creating a healthy mealtime environment	128 (69)	71 (72)	57 (65)	.26
Using positive feeding practices	127 (68)	72 (73)	55 (63)	.11
My services policies on child nutrition	130 (70)	76 (78)	54 (61)	.02
Frequency of leading planned healthy eating learning experiences				
1 time per week or more	81 (43)	48 (49)	33 (38)	.12
2–3 times per month	74 (40)	34 (35)	40 (45)	.13
1 time per month	22 (12)	15 (15)	7 (8)	.12
Rarely or never	9 (5)	1 (1)	8 (9)	.01^b^
Frequency of enthusiastic role modelling eating healthy foods served at meal and snack times				
Every meal and snack time	87 (47)	47 (48)	40 (45)	.73
Often	74 (40)	41 (42)	33 (38)	.55
Sometimes	23 (12)	10 (10)	13 (15)	.35
Rarely or never	2 (1)	0 (0)	2 (2)	n/a

^a^ *p*‐values calculated using Chi‐squared test.

^b^ *p*‐value calculated using Fisher's Exact test.

Table 3 compares the provision of nutritional guidelines to families between educators who completed PD on child nutrition ≥1 time per year (*n* = 137) and those who completed PD <1 time per year or never (*n* = 37). A significantly higher proportion of educators who completed PD <1 time per year or never, did not provide families with nutrition guidelines or resources compared to educators who completed PD ≥1 time per year (11% vs. 2%, *p* = .02). The results demonstrate that *Munch & Move* eLearning training and regular PD significantly enhanced the provision of nutrition information and practices amongst FDC educators.

**TABLE 3:** Responses from educators *n* (%) regarding the provision of nutritional guidelines to families.

Responses regarding the provision of nutritional guidelines	All educators^a^ (*n* = 174) *n* (%)	Educators who completed professional development on child nutrition ≥1 time per year (*n* = 137) n (%)	Educators who completed professional development on child nutrition <1 time per year or have never completed professional development (*n* = 37) *n* (%)	*p*‐Value^b^
Both the service provider (scheme) and I provide nutrition guidelines or resources to families	99 (57)	83 (61)	16 (43)	.06
I provide nutrition guidelines or resources to families	39 (22)	32 (23)	7 (19)	.57
The service provider (scheme) provides nutrition guidelines or resources to families	26 (15)	20 (15)	6 (16)	.81
Families are not provided with nutrition guidelines or resources	6 (3)	2 (2)	4 (11)	.02^c^

^a^ Educators who provided all food to children were excluded from this survey question.

^b^ *p*‐values calculated using Chi‐squared test.

^c^ *p*‐value calculated using Fisher's Exact test.

### 3.2 Physical activity practices

The physical activity practices examined included physical activity, outdoor playtime, outdoor play environment and education and development. Table 4 compares physical activity practices between educators trained in *Munch & Move* eLearning relating to children's physical activity (*n* = 75) and those who were not trained (*n* = 111). A significantly higher proportion of trained educators offered information on child physical activity topics to families. In regard to the amount of time provided for children's indoor and outdoor physical activity, there was no difference between groups. Less than two‐thirds of educators in both groups (57% amongst trained educators trained, 61% amongst educators not trained) met the recommendation of 90 min or more per day. The results demonstrate that *Munch & Move* eLearning training enhances the dissemination of information on child physical activity to families. However, provision of children's indoor and outdoor physical activity time is suboptimal, regardless of *Munch & Move* training background.

**TABLE 4:** Physical activity and screen time practices by educators trained in *Munch & Move* eLearning relating to children's physical activity and screen time.

	All educators (*n* = 186) *n* (%)	Educators trained in Munch & Move eLearning (*n* = 75) *n* (%)	Educators not trained in Munch & Move eLearning (*n* = 111) *n* (%)	*p*‐Value^a^
Information offered to families on children's physical activity and screen time (multiple responses allowed)				
Recommended amounts of daily physical activity for children	113 (61)	60 (80)	53 (48)	<.001
Encouraging children's physical activity	136 (73)	62 (83)	74 (67)	.02
Limiting long periods of seated time for children	95 (51)	51 (68)	44 (40)	<.001
Children's motor skill development	126 (68)	59 (79)	67 (60)	.009
Recommended amounts of outdoor playtime for children	99 (53)	55 (73)	44 (40)	<.001
Using the outdoors to encourage children's physically active play	122 (66)	60 (80)	62 (56)	<.001
My services policies on physical activity	111 (60)	54 (72)	57 (51)	.005
Recommended amounts of screen time for young children	100 (54)	51 (68)	49 (44)	.001
Appropriate types of programming for young children	98 (53)	49 (65)	49 (44)	.005
Appropriate supervision and use of screen time by caregivers	95 (51)	50 (67)	45 (41)	<.001
My services policies on screen time	104 (56)	51 (68)	53 (48)	.006

The amount of time provided for children's indoor and outdoor physical activity each day	All educators (*n* = 184)^b^	Educators trained in Munch & Move eLearning (n = 75)	Educators not trained in Munch & Move eLearning (*n* = 109)	*p*‐Value
Less than 60 min	13 (7)	6 (8)	7 (6)	.68
60–74 min	46 (25)	19 (25)	27 (25)	.93
75–89 min	16 (9)	7 (9)	9 (8)	.80
90 min or more	109 (59)	43 (57)	66 (61)	.66

The amount of screen time allowed for children 2 years of age and older	All educators (*n* = 184)^c^	Educators trained in Munch & Move eLearning (*n* = 75)	Educators not trained in Munch & Move eLearning (*n* = 109)	*p*‐Value
	*n* (%)	*n* (%)	*n* (%)	
Less than 30 min or no screen time is allowed	139 (76)	61 (81)	78 (72)	.13
30–59 min	37 (20)	10 (13)	27 (25)	.065
60–89 min	6 (3)	3 (4)	3 (3)	.69^e^
90 min or more	2 (1)	1 (1)	1 (1)	1^e^

The amount of screen time allowed for children under 2 years of age	All educators (*n* = 138)^d^	Educators trained in Munch & Move eLearning (*n* = 54)	Educators not trained in Munch & Move eLearning (*n* = 84)	*p*‐Value
	*n* (%)	*n* (%)	*n* (%)	
No screen time is allowed	79 (57)	34 (63)	45 (54)	.28
1–29 min	44 (32)	15 (28)	29 (35)	.41
30–59 min	14 (10)	4 (7)	10 (12)	.57^e^
60 min or more	1 (1)	1 (2)	0 (0)	.39^e^

^a^ *p*‐values calculated using Chi‐squared test.

^b^ Educators who provided less than 4 h of care per day were excluded from this survey question.

^c^ Educators who did not look after children 2 years of age or older were excluded from this survey question.

^d^ Educators who did not look after children under 2 years of age were excluded from this survey question.

^e^ *p*‐value calculated using Fisher's Exact test.

### 3.3 Screen time practices

The screen time practices examined included amount of sedentary screen time and education and development. Table 4 compares screen time practices between educators trained in *Munch & Move* eLearning relating to children's screen time (*n* = 75) and those who were not trained (*n* = 111). A significantly higher proportion of trained educators offered information on screen time topics to families. In regard to screen time allowed for children, there was no statistically significant difference between groups in meeting screen time guidelines. This included the guidelines of less than 30 min or no screen time for children 2 years and older (81% amongst trained educators vs. 72% amongst educators not trained) and no screen time for children under 2 years (63% amongst trained educators vs. 54% amongst educators not trained).

Table 5 compares the provision of screen time during meal or snack times between educators who completed PD on child nutrition ≥1 time per year (*n* = 144) and those who completed PD <1 time per year or never (*n* = 42). A smaller proportion of educators who completed PD <1 time per year or never responded that they sometimes have televisions or videos (5% vs. 17%). This difference was statistically significant. The results demonstrate that *Munch & Move* eLearning training significantly improves provision of screen time information to families. However, provision of sedentary screen time is suboptimal, regardless of *Munch & Move* training background.

**TABLE 5:** Responses from educators regarding the use of screen time during meal or snack times.

Responses regarding whether televisions or videos are on during meal or snack times	All educators (*n* = 186) n (%)	Educators who completed professional development on child nutrition ≥1 time per year (*n* = 144) n (%)	Educators who completed professional development on child nutrition <1 time per year or have never completed professional development (*n* = 42) n (%)	*p*‐Value^a^
Always on	0 (0)	0 (0)	0 (0)	n/a
Often on	2 (1)	1 (1)	1 (2)	.40
Sometimes on	27 (15)	25 (17)	2 (5)	.046
Never on	157 (84)	118 (82)	39 (93)	.096

^a^ *p*‐values calculated using Chi‐squared test.

## 4 DISCUSSION

This study investigated the nutrition, physical activity and screen time practices of NSW FDC educators and possible associations between *Munch & Move* training, PD and healthy practices. A higher proportion of educators who completed training in the *Munch & Move* program offered nutrition, physical activity and screen time information to families, and led planned healthy eating learning experiences for children. For the educators who had never completed PD or had completed PD <1 time per year, a higher proportion did not provide families with nutrition guidelines or resources. Although there was no statistically significant difference between *Munch & Move* trained and non‐trained groups in meeting physical activity guidelines for children, less than two‐thirds of educators in both groups did not meet the guideline of 90 min or more per day. Similarly, over one‐third of educators in both groups did not meet the guideline of no screen time for children under 2 years of age.

In examining whether educators who have been trained in the *Munch & Move* program had better nutrition practices compared to educators who have never received this training, it was found that more educators trained in *Munch & Move* offered information to families regarding serving sizes for children and information to families on the services' policies on child nutrition, compared to those who had not received this training. For educators who completed PD <1 time per year or had never completed PD, a higher proportion did not provide families with nutrition guidelines or resources. A NSW observational study found that food provision to children in FDC only met dietary recommendations for fruit, and discretionary food provision exceeded recommendations. Healthy food provisional scores were also higher in FDC services that had completed nutrition PD in the last 2 years.^10^ These results are similar to another study conducted in NSW that found an implementation intervention promoting healthy eating was able to improve policy and practice in childcare services. This implementation intervention consisted of key strategies including identifying leaders and obtaining their support and endorsement, the provision of professional development for staff, resource provision, and performance monitoring and feedback. It was determined that services where this intervention was implemented were significantly more likely to engage parents in nutrition policy or programs, to have a nutrition policy on foods brought from home, and food menus were more compliant with healthy eating guidelines.^25^ A review of childcare facilities as health promotion settings suggested opportunities for improvement in caregivers' promotion of children's health behaviours, and indicated that future interventions in child‐care settings can help improve provision of healthy food.^26^ A collaborative health promotion approach is extremely important in creating a supportive healthy environment for individuals, including collaboration with families. This ensures that the services, educators, staff, parents, carers, families and children are involved in developing an environment that will support children's healthy eating and physical activity. Collaborative partnership with families is part of the NQS.^27^ There is a high variation in the implementation of *Munch & Move* in FDC settings. This is largely due to the unique nature of FDC, where educators work independently, operating out of their homes and are likely to have more personal relationships with the families accessing their service.^15^ For *Munch & Move* to be successful, flexibility is required to allow the program to be adjusted to the cultural, socio‐economic, structural and participant profile. Collaboration between educators, families, local health authorities, and community organisations is essential to tailor the program effectively.

Our study found that more educators trained in *Munch & Move* offered physical activity and screen time information to families than educators who were not trained in *Munch & Move*. Previous studies have shown that parent attitudes, behaviours, parenting styles and practices greatly influence children's physical activity behaviours.^28^, ^29^, ^30^ It has been found that children of parents who received information on how, when and where to encourage their child's physical activity, spent more time engaging in outdoor activity compared to children whose parents received no information.^30^ Parents are important to engage with as they are aware of their children's own barriers to physical activity and can create opportunities that may be tailored to their own children's preferences.^31^ A study conducted in NSW examining the impact of an intervention to improve the adoption of physical activity policies and practices in centre based childcare services found that a significantly greater number of the intervention services had a written physical activity policy (including reference to provision of screen time) and had staff trained in physical activity compared to control services.^32^ Similarly, another study conducted in the USA found that state‐wide training to increase childcare providers' knowledge of nutrition and physical activity resulted in improvements in knowledge of state‐wide regulation components related to healthy behaviours in both centre‐based and home‐based services.^33^ This further highlights the importance of a collaborative health promotion approach where parents and families are actively involved in supporting healthy environments and suggests the importance of an intervention or program in FDC services to improve practices on a provider level, such as *Munch & Move*. By empowering educators with the necessary skills and knowledge, and fostering strong partnerships with families, *Munch & Move* can create consistent, supportive environments that promote healthy behaviours in children both at home and in childcare settings.

The *Munch & Move* program is strongly aligned with the NQF and the NQS,^18^ specifically related to ‘Quality Area 2—Children's Health and Safety’. This quality area highlights the importance of the provision of nutrition and physical activity through planned and spontaneous experiences, and role modelling healthy behaviours. Role modelling healthy eating is one of the bigger influences on a child's diet quality, yet less than half of FDC educators are doing this at every meal and snack time.^34^ Our study found that only 1% of educators trained in *Munch & Move* responded that they rarely or never led planned healthy eating learning experiences compared to 9% of educators who were not trained. Active learning experiences ensure that young learners can master, generalise and retain what they learn.^35^ The positive effects of active learning experiences on willingness to try new foods and on dietary intake have been demonstrated in a number of studies. For example, a study conducted in Sweden found that when food was used as a tool for planned learning experiences, children were more positive to new food tastings and developed a sensory language.^36^ An Australian study found an association between intentional healthy eating learning experiences in childcare centres and reduced intake of saturated fat.^37^ Our findings support the potential benefits of *Munch & Move* training in promoting healthier nutrition practices through encouraging educators to lead active learning experiences.

The train‐the‐trainer aspect of *Munch & Move* has played a crucial role in the variability of training outcomes. Previously, *Munch & Move* training relied heavily on service providers to train educators. This approach was ad‐hoc and subject to personal interpretation and modification, leading to inconsistencies in training both between and within FDC services. The reliance on service providers meant that the quality of training could vary significantly, depending on the providers' confidence, capability and clarity in communicating key concepts. To address these inconsistencies, *Munch & Move* has since modified its eLearning training to be self‐paced and offered directly to educators. This will reduce the previous inconsistencies by ensuring all participants received the same information about the program. Educators now have increased ownership over how they implement *Munch & Move*, which may explain why educators who participated in the new eLearning training exhibited better practices in several areas in the present study. However, service providers still play a significant role in driving program implementation and ongoing engagement from their educators. Therefore, it may be beneficial to create additional opportunities for engaging service providers and build a community of practice.

We found suboptimal provision of children's indoor and outdoor physical activity time, regardless of *Munch & Move* training background. In both groups, less than two‐thirds met the guidelines of providing children with 90 min or more per day of physical activity time.^38^ A systematic review investigating the physical activity levels of children aged 0–6 years old in FDC services found that the average moderate‐ to vigorous‐intensity activity time and total physical activity time was 5.8 and 10.4 min per h, respectively.^39^ An Australian audit of FDC services also found that the average moderate‐ to vigorous‐intensity activity time and total physical activity time was only 2.1 and 3.57 min per h, respectively.^12^ Similar to our results, this is lower than the national guidelines.^39^ In addition, we found that over one third of educators in both groups did not meet the guideline of no screen time for children under 2 years of age.^39^ A systematic review of screen time amongst pre‐schoolers in childcare found that children engaged in 108–114 min of screen time per day.^40^ Similarly, our findings do not meet the guidelines for screen time. Early childhood is a critical period for developing healthy habits, and insufficient physical activity coupled with excessive screen time can have implications on children's physical, cognitive and social development.^1^ However, not all children have the opportunity at home to develop healthy lifestyle behaviours.^41^ This makes ECEC settings extremely important in providing those opportunities to children to improve or support behaviours learned at home. Given the amount of time children spend in FDC settings (for example, 84% of educators in this study stated that they provide 7–10 h of care per day to children), ensuring adherence to physical activity and screen time guidelines is critical.

There are limitations to the present study. Self‐reported data may be subject to reporting bias and social desirability bias.^42^ Educators who were trained in *Munch & Move* may have known the expected answers to the survey questions. The present study is cross‐sectional and therefore causality cannot be established. Convenience sampling can introduce selection bias, potentially skewing results. The study focuses on FDC educators in NSW, limiting the applicability of findings to other regions with different demographic and regulatory contexts. Future research could incorporate objective measures, such as direct observations, or conduct randomised controlled trials to help control potential biases and examine the efficacy of *Munch & Move*.

The findings from this study have several important implications for policy and practice. The findings highlight the crucial role of training and PD in promoting healthy practices in FDC settings. While *Munch & Move* training has shown positive effects, there are still areas for improvement. The results highlight physical activity and screen time as areas for improvement where further training can be provided. This can involve creating additional opportunities to regularly engage the FDC sector, including informal engagement, to build the sense of community and foster learning experiences. Utilising support from Local Health Districts, particularly with ideas to inspire educators, additional PD opportunities and personalised resources can also be valuable. Workshops focused on practical ideas for integrating physical activity into daily routines can be beneficial. Given the amount of time children spend in FDC settings, ensuring adherence to guidelines is critical to improve or support healthy behaviours. Since *Munch & Move* trained educators are more likely to provide information to families, there is an opportunity to engage parents in promoting healthy behaviours at home. Strategies to enhance parent‐educator communication and collaboration, ensuring that health promotion efforts are reinforced both in FDC settings and at home, may be beneficial.

## 5 CONCLUSION

This study demonstrates the potential benefits of the *Munch & Move* program and PD. FDC educators trained in the *Munch & Move* program, and those who completed PD more frequently, demonstrated better nutrition, physical activity and screen time practices in several areas. These included offering information on nutrition, physical activity and screen time to families, and leading planned learning experiences related to healthy behaviours. The results highlight physical activity and screen time as areas for improvement where further training can be provided. Individual and environmental factors such as educator knowledge and confidence and available outdoor space may also contribute to the suboptimal provision of physical activity and screen time. Future research investigating this area may be worthwhile.

## AUTHOR CONTRIBUTIONS

All authors have contributed to the present paper through involvement in the conception and design of the study or in analysis and interpretation of the data. All authors were involved in writing or revising the paper and have approved the final article.

## FUNDING INFORMATION

This work was supported by the Prevention Research Support Program, funded by the New South Wales Ministry of Health.

## CONFLICT OF INTEREST STATEMENT

The authors have no conflict of interest to declare.

## ETHICS STATEMENT

This study was conducted according to the guidelines laid down in the Declaration of Helsinki, and all procedures involving research participants were approved by the University of Wollongong Human Research Ethics Committee (HREC/18/WGONG/13). Online informed consent was obtained from all educators of participating services.

## Data Availability

The data that support the findings of this study are available from the corresponding author upon reasonable request.
